# An integrated transcriptome and epigenome analysis identifies a novel candidate gene for pancreatic cancer

**DOI:** 10.1186/1755-8794-6-33

**Published:** 2013-09-22

**Authors:** Jinping Jia, Hemang Parikh, Wenming Xiao, Jason W Hoskins, Holger Pflicke, Xuelu Liu, Irene Collins, Weiyin Zhou, Zhaoming Wang, John Powell, Snorri S Thorgeirsson, Udo Rudloff, Gloria M Petersen, Laufey T Amundadottir

**Affiliations:** 1 Laboratory of Translational Genomics, Division of Cancer Epidemiology and Genetics, National Cancer Institute, National Institutes of Health, Bethesda, MD 20892, USA; 2 Division of Computational Bioscience, Center for Information Technology, National Institutes of Health, Bioinformatics and Molecular Analysis Section, Bethesda, MD 20892, USA; 3 Thoracic and Gastrointestinal Oncology Branch, Center for Cancer Research, National Cancer Institute, National Institutes of Health, Bethesda, MD 20892, USA; 4 SRA International, Fairfax, VA 22033, USA; 5 Cancer Genomics Research Laboratory, Division of Cancer Epidemiology and Genetics, National Cancer Institute, National Institutes of Health, Bethesda, MD 20892, USA; 6 Laboratory of Experimental Carcinogenesis, National Cancer Institute, National Institutes of Health, Center for Cancer Research, Bethesda, MD 20892, USA; 7 Department of Health Sciences Research, Mayo Clinic, Rochester, MN 55905, USA

**Keywords:** Pancreatic cancer, Transcriptome, Epigenome, Sequencing, *ALDH1A3*

## Abstract

**Background:**

Pancreatic cancer is a highly lethal cancer with limited diagnostic and therapeutic modalities.

**Methods:**

To begin to explore the genomic landscape of pancreatic cancer, we used massively parallel sequencing to catalog and compare transcribed regions and potential regulatory elements in two human cell lines derived from normal and cancerous pancreas.

**Results:**

By RNA-sequencing, we identified 2,146 differentially expressed genes in these cell lines that were enriched in cancer related pathways and biological processes that include cell adhesion, growth factor and receptor activity, signaling, transcription and differentiation. Our high throughput Chromatin immunoprecipitation (ChIP) sequence analysis furthermore identified over 100,000 regions enriched in epigenetic marks, showing either positive (H3K4me1, H3K4me3, RNA Pol II) or negative (H3K27me3) correlation with gene expression. Notably, an overall enrichment of RNA Pol II binding and depletion of H3K27me3 binding were seen in the cancer derived cell line as compared to the normal derived cell line. By selecting genes for further assessment based on this difference, we confirmed enhanced expression of aldehyde dehydrogenase 1A3 (*ALDH1A3*) in two larger sets of pancreatic cancer cell lines and in tumor tissues as compared to normal derived tissues.

**Conclusions:**

As aldehyde dehydrogenase (ALDH) activity is a key feature of cancer stem cells, our results indicate that a member of the ALDH superfamily, *ALDH1A3*, may be upregulated in pancreatic cancer, where it could mark pancreatic cancer stem cells.

## Background

Although pancreatic cancer is the tenth most commonly diagnosed cancer in the U.S., it is the fourth most common cause of cancer mortality with close to 37,000 deaths per year in the U.S. [1]. The aggressive nature of this cancer is further emphasized by the dismal five-year survival rate of less than 5% [2]. Pancreatic ductal adenocarcinoma (PDAC), the most common form of pancreatic cancer, is thought to arise through a multistep process involving intermediate precursor lesions known as pancreatic intraepithelial neoplasias (PanINs) [3]. Genome-wide sequencing approaches have revealed a complex set of somatic alterations in pancreatic tumors such as single base mutations, amplifications, deletions and complex rearrangements that drive cancerous growth through specific signaling pathways [4,5]. The genes most frequently altered in pancreatic cancer are *KRAS*, *TP53*, *CDKN2A* and *SMAD4*, leading to an activation of growth promoting and cell survival pathways, and inactivation of tumor suppressors and apoptotic pathways [4].

In addition to an accumulation of somatic mutations that affect the DNA sequence directly, epigenetic events can lead to an increase in cell growth or survival and thereby contribute to tumor development [6]. DNA methylation and post-translational modification of histone proteins are epigenetic mechanisms that regulate gene expression in normal and cancerous cells. DNA methylation at specific genes can increase or decrease in cancer and is often associated with an inactivation of tumor suppressor genes [7]. Post-translational modification of histones mediates appropriate gene expression by regulating access of the transcriptional machinery to the underlying DNA [8]. Changes in DNA methylation have been investigated in pancreatic cancer [9-11] but genome wide maps of histone modification patterns have not been reported in this disease.

To enhance our understanding of the pancreatic cancer genome, we catalogued transcribed sequences and potential functional elements in cell lines derived from normal and neoplastic pancreatic tissues by next generation RNA-sequencing and chromatin immunoprecipitation (ChIP) with massively parallel sequencing. Differentially expressed genes and pathways were identified, and gene expression was correlated to histone modification patterns that mark active and repressed chromatin.

## Methods

### Cell lines and cell culture

Two human pancreatic cell lines were purchased from ATCC: hTERT-HPNE (derived from normal ductal pancreatic tissue, immortalized with the *TERT* gene; modal chromosome number = 46) [12,13] and PANC-1 (derived from pancreatic ductal adenocarcinoma; modal chromosome number = 63) [14]. The hTERT-HPNE cell line was cultured in 75% DMEM (ATCC), 25% Medium M3 Base (Incell Corporation), supplemented with 5% fetal bovine serum (FBS), 10 ng/ml human recombinant EGF, 5.5 mM D-glucose (1 g/L) and 750 ng/ml puromycin. The PANC-1 cell line was cultured in DMEM (ATCC) supplemented with 10% FBS. Cells were grown to log phase and harvested for RNA and ChIP sequencing experiments at approximately 60-80% confluency. The cell lines were harvested at passage 18 (PANC-1) and 23 (hTERT-HPNE) after purchase from ATCC.

### RNA preparation

Total RNA was isolated from ~5×10^6^ cells using the mirVana™ miRNA isolation kit (Ambion) according to the manufacturer’s protocol (retaining the small RNA fraction in the pool of total RNA). DNAse treatment was performed with TURBO DNase (Ambion); RNA quantity was assessed by Nano Drop (Thermo Scientific) and RNA quality by using the Agilent 2100 Bioanalyzer (Agilent Technologies). RNA quality (RIN) scores for RNA prepared from cell lines were > 9.0.

### ChIP and template preparation

Chromatin immunoprecipitation (ChIP) was performed with the ChIP-IT™ Express kit (Active Motif) according to the manufacturer’s protocol. Briefly, cells were grown to log phase and cross-linked with 1% formaldehyde for 10 minutes at room temperature. The fixation reaction was stopped by a glycine stop solution and cells were washed in PBS and homogenized in lysis buffer supplemented with protease inhibitors. Shearing was performed by sonication in a Diagenode Bioruptor (30-sec on/off pulses for 15–20 min at high setting) to obtain 200–500 bp DNA fragments. After centrifugation, the supernatant was used for chromatin immunoprecipitation (from ~1×10^7^ cells each) using antibodies for: anti-mono-methylated histone H3 at lysine 4 (H3K4me1) (Abcam, ab8895), anti-tri-methylated histone H3 at lysine 4 (H3K4me3) (Abcam, ab12209), anti-tri-methylated histone H3 at lysine 27 (H3K27me3) (Abcam, ab6002) and anti-RNA polymerase II (Pol II) (Abcam, ab817). Input samples consisted of 10 μL sonicated chromatin. For each ChIP, 20 μg sheared chromatin was incubated overnight at 4°C on an end-to-end rotator, with 8μg antibody, protein G magnetic beads and protease inhibitors. Finally, the ChIP and input DNA samples were reverse cross-linked, treated with proteinase K and purified with the QIAquick PCR Purification Kit (Qiagen).

### Library preparation and sequencing

For mRNA-seq sample preparation, the mRNA-seq Sample Prep Kit (Illumina) was used according to the manufacturer’s protocol. Briefly, 1 μg total RNA was used for polyA mRNA selection using Sera-mag oligo(dT) beads, followed by thermal fragmentation at 94°C. First strand cDNA synthesis was performed using Superscript II reverse transcriptase and random primers. Second strand synthesis was performed using DNA Polymerase I followed by end repair with T4 DNA polymerase, Klenow DNA polymerase and T4 polynucleotide kinase (PNK). Finally, the double-stranded cDNA was ligated to Illumina paired-end (PE) adaptors and size selection performed for fragments in the 350±25 bp range. Libraries were amplified using Phusion DNA polymerase, followed by purification with the QIAquick PCR Purification Kit (Qiagen). Amplified libraries were diluted with Elution Buffer to a final concentration of 10 nM and run at a concentration of 4–6 pM on two Genome Analyzer (GAII) lanes.

MicroRNA-seq sample preparation was performed with the Small RNA v1.5 sample prep Kit (Illumina) according to the manufacturer’s protocol. Briefly, 3′ and 5′ small RNA adapters were ligated to 10 μg total RNA followed by reverse transcription with Superscript II and amplification using Phusion DNA polymerase. This was followed by size selection of cDNA in the 100–108 bp range and measurement using a Bioanalyzer. Amplified miRNA-seq libraries were diluted with Elution Buffer to a final concentration of 10 nM and run at a concentration of 4–6 pM on one Genome Analyzer (GAIIx) lanes.

For ChIP-seq sample preparation, pooled ChIP reactions (10 ng) were used to prepare a single library using the ChIP-seq Sample Prep Kit (Illumina) according to the manufacturer’s protocol. Briefly, DNA ends were repaired with T4 DNA polymerase, Klenow polymerase and T4 PNK. The Klenow fragment (3′ to 5′ exo minus) was then used to generate a protruding 3′ A base and adapter oligos were ligated using DNA ligase. ChIP DNA in the 200±25 bp range was recovered and amplified using Phusion polymerase. Library concentration was assessed using a Bioanalyzer. Amplified libraries were diluted with Elution Buffer to a final concentration of 10 nM and run at a concentration of 4–6 pM on 2–3 Genome Analyzer (GAII or GAIIx) lanes. All next-generation sequencing was carried out at the National Cancer Institute’s Center for Cancer Research Sequencing Facility operated by the Advanced Technology Program of SAIC-Frederick, Inc. (Gaithersburg, MD).

### Sequence alignment and analysis

*mRNA-sequencing*: paired-end RNA-seq was repeated twice for each cell line to obtain reliable numbers of sequence reads (Additional file 1: Table S1A). The median number of reads (35 bp) generated per paired-end experiment was 8.9 M (8.6 to 11.2 M). Total reads were combined for each cell line: 19,778,468 for the hTERT-HPNE and 17,769,249 for PANC-1 cells. No significant difference was observed in the number of reads generated between the two cell lines (Student’s *t*-test, *P*=0.53). Paired-end reads were mapped to the RefSeq database (National Center for Biotechnology Information (NCBI) build 37) using the Burrows-Wheeler Aligner (BWA) software with default parameters that allow up to 3 alignments for each read and up to 2 mismatches for the seed sequence (the first 25 bp of each read) [15]. Reads that failed to map to RefSeq were mapped to the Ensembl database, which includes additional transcripts and pseudogenes [16]. Remaining unmapped reads were mapped to the human genome assembly (NCBI build 37). The analysis pipeline is shown in Additional file 2: Figure S1. Gene expression was calculated in reads per kilobase of exon per million mapped sequence reads (RPKM) [17]. After filtering, we included 11,249 genes expressed above 1 RPKM in at least one cell line for further analysis. The edgeR package was used to identify genes that were differentially expressed in the two cell lines using total tag count values for each gene [18]. Data was normalized using the weighted trimmed mean of log-ratio values (using hTERT-HPNE as a reference) to account for library size; statistical analysis was performed using the Fisher’s exact test. We considered genes to be differentially expressed between cell lines when the Benjamini-Hochberg false discovery rate (FDR) was less than 0.05 and the fold change difference in expression was equal to or greater than 3.

*miRNA-sequencing*: After trimming adaptor sequences and filtering low-quality reads, a total of 9,262,094 and 18,006,865 single-end short (22 bp) sequence reads were obtained from PANC-1 and hTERT-HPNE cells, respectively (Additional file 1: Table S1B). The miRNAkey software [19] was used to remove adaptor sequence from the 3′-ends of reads, to map raw reads to the miRBase mature database (release 16) [20], calculate normalized RPKM expression values and quantify differentially expressed miRNAs. Two hundred and fifty eight miRNA genes were detected at 1 RPKM or higher in at least one of the cell lines. Genes were considered differentially expressed with a fold change difference ≥ 3 and a Bonferroni corrected Chi-square *P*-value < 0.05.

*Pathway enrichment analysis*: Gene set enrichment analysis of differentially expressed mRNA and miRNA genes was performed based on KEGG (Kyoto Encyclopedia of Genes and Genomes) [21] and GO (Gene Ontology) [22] annotations using GOseq, as it accounts for the bias of over-detection of differential expression for long and highly expressed transcripts [23]. The Wallenius approximation method with Benjamini-Hochberg corrected *P*-values was used to determine enriched pathways; and an FDR < 0.05 was considered significant.

*ChIP-sequencing:* ChIP-seq single-end experiments were run on 2–3 lanes to obtain an adequate number of reads (Additional file 1: Table S1C). Matching input DNA was sequenced to obtain a reference for each cell line. The total number of short (35 bp) reads generated per lane varied from 4,509,035 to 27,577,446 with a median of 19,279,525. Raw reads were combined for each histone modification mark and RNA Pol II as well as for input DNA for each of the cell lines. No significant difference was observed in the number of reads generated in the two cell lines for the different histone modification marks or for RNA Pol II (Student’s *t*-test, *P*-value range: 0.17-0.83). Alignment to the human genome assembly (NCBI build 37) was performed using Bowtie, allowing for up to 2 mismatches and only one (best) alignment [24]. The SICER software [25] was used to identify qualified peaks (islands) of histone and Pol II binding by comparing sequence reads from immunoprecipitated and input DNA. A consolidating window size of 200 bps was used, a gap size of 200 bps (H3K4me1, H3K4me3 and Pol II) or 600 bps (H3K27me3), effective genome size of 81%, ratio of enrichment between experimental data (PANC-1) and control (hTERT-HPNE) ≥3 and an FDR <0.05. Differentially enriched epigenetic marks across the two cell lines were identified by the SICER-df function. A cis-regulatory element annotation system (CEAS) was used to attain summary statistics on ChIP enrichment peaks based on location in promoters, gene bodies or nongenic regions using the RefSeq database [26].

### Integration of ChIP-seq and RNA-seq

Genes were divided into quartiles based on digital expression levels (RPKM values) for each cell line. Global profiling curves were generated for genes in each of the quartile groups by plotting the read distributions (tags were binned into 25 bps bins and trimmed based on Poisson distribution) of different histone modification marks and RNA Pol II within 5,000 bp up- and downstream of transcription start sites (TSS) for RefSeq genes.

### Genomic copy number alterations

To assess if a bias existed in read mapping for ChIP-Seq because of chromosomal copy number gains or losses, we extracted DNA (Gentra Puregene Tissue Kit, Qiagen) from the hTERT-HPNE and PANC-1 cell lines, and genotyped at NCI’s Cancer Genomics Research Laboratory using the Illumina Human Hap 1M-Duo chip. The Illumina GenomeStudio software was used to compute affinity-normalized probe intensities (normalized genotype probe intensities, log R ratio (LRR) and B allele frequency (BAF)), quality scores and to call genotypes. Renormalized (quantile) LRR and BAF values were then analyzed using a custom software pipeline, R-Gada [27], to detect whole-chromosome and segmental events as previously described [28].

We only observed chromosomal copy number alterations in the PANC-1 cell line but not in the hTERT-HPNE cells. Adjacent events were merged if they had an identical state and distance between segments was <1 Mb. Each event was then classified as copy number gain, loss or copy neutral loss of heterozygosity (CNLOH). Since we observed only 200 (0.49%) regions enriched for RNA Pol II binding and 41 (0.57%) regions enriched for H3K27me3 binding in regions that were lost in PANC-1 cells, these were excluded from further analysis. To estimate a possible bias in mapping, we then calculated the number of regions enriched for RNA Pol II and H3K27me3 binding in the PANC-1 cells per 1 Mb for each autosomal chromosome for gain, CNLOH and copy neutral events. For interclass, unpaired comparisons, we used the Mann–Whitney U-test to assess differences in the distribution of genomic regions enriched for RNA Pol II and H3K27me3 binding in the PANC-1 cells.

To assess if genes selected for replication by TaqMan expression analysis (see below) were located in regions of copy number gain or loss, we also genotyped the following cell lines and assessed chromosomal abnormalities as described above: AsPC-1, BxPC-3, Capan-1, CFPAC-1, Hs 766T, SU.86.86 and SW 1990.

### Assessment of differential mRNA expression analysis using Real-Time qPCR

An assessment of differential expression levels was attempted for 4 genes using custom mRNA Taqman expression assays (Applied Biosystems): *ALDH1A3* (Hs00167476_m1), *ITGBL1* (Hs00191224_m1), *NFE2L3* (Hs00852569_g1) and *SEMA4B* (Hs00384240_m1). Fresh frozen pancreatic tissue samples (10 normal and 10 tumor derived) were obtained from the Mayo Clinic in Rochester MN (approved by the Institutional Review Board of the Mayo Clinic). All tumors were from patients diagnosed as adenocarcinoma of the pancreas, with tumor percentages ≥70%; all normal derived samples were adjacent to tumors (unmatched to tumors), confirmed by a pathologist to be normal by pathology and with ≥80% epithelial component. Eleven additional pancreatic cancer cell lines were purchased from ATCC and cultured according to their guidelines: AsPC-1, BxPC-3, Capan-1, Capan-2, CFPAC-1, Hs 766T, HPAF-II, MIA PaCa-2, Panc 10.05, SU.86.86 and SW 1990. An additional 39 pancreatic cancer cell lines (see Additional file 3: Table S6 for names of cell lines and references) were kindly provided by Dr. Udo Rudloff, Surgery Branch, NCI, NIH, and maintained in DMEM with 10% FBS. Frozen tissue sections (10 μm cut in at −20°C in a Cryostat) and cell lines (log phase) were harvested for RNA isolation as described above. Total RNA was reverse transcribed using Superscript III first-strand synthesis kit (Invitrogen). RT-qPCR was performed in triplicates using Taqman gene expression Master Mix (Applied Biosystems) and a 7900HT sequence detecting system (Applied Biosystems). The *Cy*_*0*_ method was used to obtain the best fit estimators of reaction parameters for real-time PCR data [29]. The ΔΔCt method was used to calculate expression values by normalizing to the geometric mean of two housekeeping genes (*B2M* #Hs00187842_m1 and *PPIA* #Hs99999904_m1; Applied Biosystems).

## Results

Using a genome-wide approach, we have assessed global gene expression profiles and regulatory elements in two cell lines derived from normal pancreatic ducts (hTERT-HPNE) and pancreatic ductal adenocarcinoma (PANC-1) by massively parallel sequencing-by-synthesis.

### Transcriptome analysis of pancreatic cell lines by mRNA-seq and miRNA-seq

The total number of paired-end mRNA-sequence reads generated for each cell line is listed in Additional file 1: Table S1A. We constructed our mRNA-seq analysis pipeline to align reads sequentially to the RefSeq, Ensembl and Human Genome (NCBI build 37) databases using BWA [15] (Additional file 2: Figure S1). By comparing the cancer derived PANC-1 cells to the normal pancreatic tissue derived hTERT-HPNE cells, we identified 1,983 differentially expressed mRNA genes at a threshold of 3-fold difference or greater (Additional file 4: Table S2). Of these genes, 971 were expressed at higher levels and 1,012 at lower levels in the cancer derived cells compared to the normal derived pancreatic cells. Examples of mRNA genes that were expressed at higher levels in the PANC-1 cell line include growth factors, receptors, and signaling molecules such as *SHH*, *PDGFB*, *SMAD3*, *AKT2* and multiple *WNT* genes (*WNT6*, *WNT7B*, *WNT9A* and *WNT10A*). Genes expressed at lower levels in the PANC-1 cells include *CDKN1A, CDKN2B*, *HHIP*, *PDGFRA*, *PDGFRB* and *MMP1*. In addition, many genes encoding extracellular matrix (ECM) proteins and their receptors (integrins) were differentially expressed in the two cell lines.

The total number of single-end miRNA sequence reads is listed in Additional file 1: Table S1B. We aligned reads to miRNA genes in the miRBase mature database, using miRNAKey [19]. Of the 258 miRNA genes expressed at 1 RPKM or higher, 128 were expressed at higher levels and 35 at lower levels in the cancer derived PANC-1 as compared to normal derived hTERT-HPNE cell line (Additional file 5: Table S3). Differential miRNA expression levels included higher levels of *MIR767, MIR135B, MIR1269, MIR182, MIR183,* and *MIR203* and lower levels of *MIR494*, *MIR424*, *MIR381*, *MIR452,* and *MIR155* in PANC-1 cells.

We used KEGG [21] and GO [22] enrichment analysis to categorize differentially expressed mRNA and miRNA genes into biologically relevant pathways and processes. The fifteen significantly enriched KEGG pathway categories included: pathways in cancer, focal adhesion, ECM-receptor interaction, Wnt signaling and cell adhesion molecules (Table 1). An enrichment of genes related to cardiomyopathy was also noted, but the list of genes overlapped considerably with two other pathways: focal adhesion and ECM-receptor interaction. Similarly, we detected significant enrichment of 56 GO biological process categories including: signal transduction, cell differentiation and cell adhesion (Additional file 6: Table S4). We also detected 25 GO molecular function categories including: signal transducer activity, receptor activity, transcription factor activity, calcium ion binding, actin binding and growth factor activity (Additional file 7: Table S5).

**Table 1:** KEGG pathway enrichment analysis for mRNA and miRNA genes differentially expressed in tumor and normal derived pancreatic cell lines

**KEGG pathway ID**	**KEGG pathway name**	***P*-value**	**FDR**	**# of genes**
05200	Pathways in cancer	5.40 × 10^-06^	1.15 × 10^-04^	69
04510	Focal adhesion	1.05 × 10^-11^	1.12 × 10^-09^	61
04512	ECM-receptor interaction	1.11 × 10^-14^	2.37 × 10^-12^	36
04060	Cytokine-cytokine receptor interaction	3.43 × 10^-07^	1.22 × 10^-05^	36
04310	Wnt signaling pathway	6.26 × 10^-05^	8.75 × 10^-04^	36
04360	Axon guidance	3.82 × 10^-06^	9.05 × 10^-05^	33
04514	Cell adhesion molecules (CAMs)	2.27 × 10^-08^	9.67 × 10^-07^	29
04020	Calcium signaling pathway	1.47 × 10^-05^	2.61 × 10^-04^	29
04080	Neuroactive ligand-receptor interaction	1.78 × 10^-08^	9.47 × 10^-07^	26
05412	Arrhythmogenic right ventricular cardiomyopathy (ARVC)	1.44 × 10^-08^	9.47 × 10^-07^	24
05414	Dilated cardiomyopathy	3.82 × 10^-06^	9.05 × 10^-05^	22
05410	Hypertrophic cardiomyopathy (HCM)	2.64 × 10^-06^	8.02 × 10^-05^	21
04640	Hematopoietic cell lineage	1.47 × 10^-05^	2.61 × 10^-04^	16
05217	Basal cell carcinoma	6.58 × 10^-05^	8.75 × 10^-04^	15
04610	Complement and coagulation cascades	1.01 × 10^-04^	1.27 × 10^-03^	10

*KEGG* pathway ID numbers are listed and statistical significance is shown by *P* values and FDR values.

### Epigenome analysis of normal and cancer derived pancreatic cell lines by ChIP-seq

To evaluate genome-wide distribution of epigenetic marks that associate with active or repressed expression, we performed ChIP-seq in the PANC-1 and hTERT-HPNE cell lines using antibodies specific for three histone modification marks: H3K4me1, H3K4me3 and H3K27me3, and for RNA Pol II. Reads (Additional file 1: Table S1C) were aligned to the human genome (NCBI build 37) using Bowtie [24]. Binding sites for histone modification marks and RNA Pol II were identified by comparing immunoprecipitated chromatin with input DNA using SICER [25]. The total number of all binding sites for H3K4me1, H3K4me3, H3K27me3, and RNA Pol II identified in each cell line at a FDR of ≤0.001 was 101,797 for the PANC-1 cell line and 115,371 for the hTERT-HPNE cell line (Additional file 1: Table S1C).

The most prominent binding sites in promoters (up to 3 kb upstream of known transcription start sites) were seen for H3K4me3 (26.6% of all sites in the hTERT-HPNE cell line and 27.3% in the PANC-1 cell line), in accord with its role in activation of gene expression. A similar pattern of binding sites was seen for H3K4me3 and RNA Pol II in the two cell lines, particularly in exons (range 2.7-3.1%), untranslated regions (range 2.8-4.0%) and introns (41.2-41.4% for RNA Pol II and 49.2-51.9% for H3K4me3). Similar overall binding patterns were noted for H3K4me1 and H3K27me3 in both cell lines with regard to promoters (4.1-7.6%), exons (0.9-1.8%) and untranslated regions (0.7-1.4%). A greater number of intronic binding sites were seen for H3K4me1 (54.3-56.7%) as compared to H3K27me3 (36.8-38.1%), and fewer sites were noted in distal intergenic regions for H3K4me1 (28.9-37.6%) as compared to H3K27me3 (50.5-51.5%) (Additional file 8: Figure S2).

### Integration of ChIP-seq and mRNA-seq

**Figure 1:**
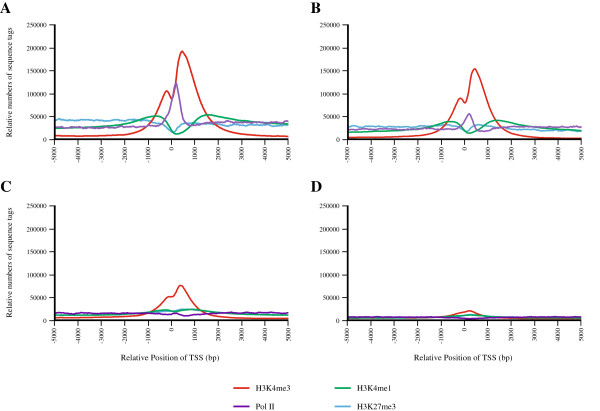
**Distribution of histone modification marks and RNA Polymerase II binding sites according to gene expression levels for RefSeq genes.** Genes were divided into four categories according to gene expression values in RPKM from high **(A)**, medium **(B)**, low **(C)** to very low **(D)**. Density traces for histone modification sequence tags were then graphed according to the start of transcription (TSS) for each of the four groups. Density traces are labeled in green for H3K4me1, red for H3K4me3, blue for H3K27me3 and purple for RNA Pol II (see panel insert). Results are shown for the PANC-1 cell line; similar results were seen for the hTERT-HPNE cell line (Additional file 9: Figure S3).

**Figure 2:**
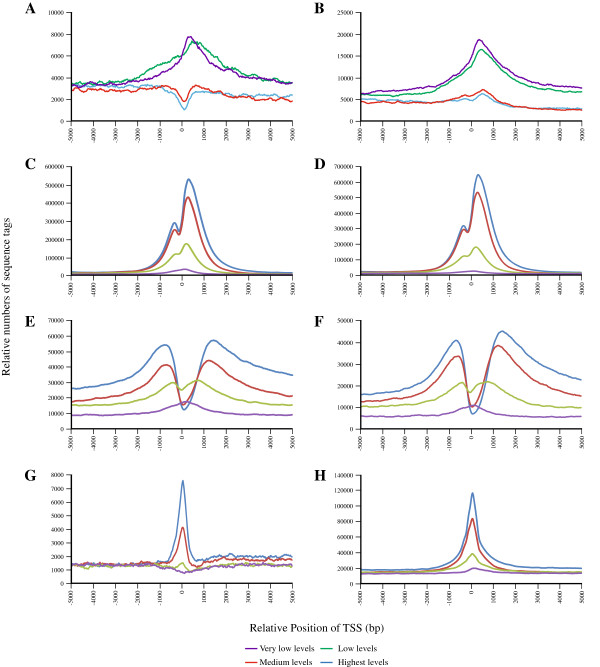
**Distribution of the histone modification marks and RNA Polymerase II binding sites relative to transcriptional start sites (TSS) for RefSeq genes.** Genes were divided into four categories based on digital gene expression (RPKM values) from high to very low. Sequence reads were then graphed relative to the TSS for each group. Density traces are labeled in blue for genes expressed at the highest levels, red for medium levels, green for low levels and purple for very low levels (see panel insert). Results are shown for the PANC-1 cell line (panels **(A)** for H3K27me3, **(C)** for H3K4me3, **(E)** for H3K4me1 and **(G)** for RNA Pol II) and for the hTERT-HPNE cell line (panels **(B)** for H3K27me3, **(D)** for H3K4me3, **(F)** for H3K4me1, and **(H)** for RNA Pol II). Note the “dip” in H3K27me3 sequence tags around the TSS of highly expressed genes in PANC-1 cells.

To correlate enrichment for histone modification marks to gene expression, we divided genes into four equal subsets based on digital gene expression levels (in RPKM) from highest to lowest. We then summarized sequence reads from ChIP-seq relative to transcription start sites (TSS) (+/− 5,000 bps) for all RefSeq genes (Figure 1 for the PANC-1 cell line and Additional file 9: Figure S3 for the hTERT-HPNE cell line) to assess their patterns in promoter elements of genes expressed at different levels. Read density for H3K4me1 and H3K4me3 was increased surrounding the TSS of highly expressed genes in both cell lines, especially for the latter histone mark. The distribution of reads is bimodal, with an increased number of reads upstream and downstream of the TSS and a valley in-between. The peaks are located at approximately 600–800 bps upstream and 1,200-1,400 bps downstream of the TSS for H3K4me1 and at approximately 300–400 bps up- and downstream of the TSS for H3K4me3 with the latter being more prominent. RNA Pol II binding sites were prominent over highly expressed genes with a peak of greatest density right around the TSS. Genes expressed at lower levels gradually lost binding of histones modified at lysine 4 and RNA Pol II (Figure 1, Figure 2, Additional file 9: Figure S3). In contrast, these genes are characterized by a relatively high density of reads for H3K27me3 with the greatest enrichment at approximately 200–300 bps downstream of the TSS (Figure 2A and B). Notably, when comparing epigenetic marks across the two cell lines, the greatest fraction of peaks enriched ≥3 fold in the PANC-1 cells was seen for RNA Pol II (71.8% increased, 28.2% decreased) and the greatest number of peaks decreased ≥3 fold in the PANC-1 cells was seen for the negative H3K27me3 histone modification mark (81.0% decreased, 19.0% increased), implying that the cancer cells may be better poised for active transcription (Table 2A). This appears to happen on a genome wide level and can be seen in Figure 2 where H3K27me3 sequence tags “dip” around the TSS of highly expressed genes in the PANC-1 cell line (Figure 2A) but not the hTERT-HPNE line (Figure 2B).

**Table 2:** Characteristics of epigenetic marks that were increased or decreased over 3 fold in the PANC-1 as compared to the hTERT-HPNE cell line

**A. Epigenetic marks that were enriched or depleted in PANC-1 as compared to hTERT-HPNE pancreatic cells**					
		**Increased in PANC-1 cells**		**Decreased in PANC-1 cells**	
**Mark**	**Fold change**	**# Increased**	**% Increased**	**# Decreased**	**% Decreased**
RNA Pol II	≥ 3	42,809	71.8%	16,841	28.2%
H3K4Me3	≥ 3	4,434	53.7%	3,828	46.3%
H3K4me1	≥ 3	13,999	26.1%	39,556	73.9%
H3K27Me3	≥ 3	2,474	19.0%	10,521	81.0%
**B. Differential epigenetic marks (change ≥ 3 fold) in the *ALDH1A3 * gene in PANC-1 and hTERT-HPNE pancreatic cell lines**					
		**RNA Pol II**		**H3K27me3**	
**Gene symbol**		*ALDH1A3*		*ALDH1A3*	
**Chromosome**		chr15		chr15	
**Start (bp)**		101,420,000		101,435,600	
**End (bp)**		101,420,199		101,439,799	
**PANC-1 readcount**		1.119		1.181	
**hTERT-HPNE readcount**		0.065		5.404	
**Fold change**		12.39		0.23	
***P*value**		1.08 x 10^-13^		8.55 x 10^-24^	
**FDR**		3.16 x 10^-13^		1.27 x 10^-22^	

A. Number and percentages of epigenetic marks that were increased or decreased over 3 fold in the PANC-1 as compared to the hTERT-HPNE cell lines.

B. Epigenetic marks in the *ALDH1A3* gene are listed with location (NCBI build 37) of the respective epigenetic mark, normalized read counts, fold change and significance values (*P* value and False Discovery Rate, FDR).

To verify that this differential enrichment in RNA Pol II and H3K27me3 binding sites was not caused by a bias in read mapping due to differences in DNA copy number, we genotyped the cell lines using the Illumina Human Hap 1M chip. We noted chromosomal alterations in the PANC-1 cells but not in the hTERT-HPNE cells. To assess mapping bias, we compared binding sites for RNA Pol II and H3K27me3 in genomic regions in the PANC-1 cells showing either gain or copy neutral loss of heterozygosity (CNLOH) relative to copy neutral genomic regions. We did not observe statistically significant differences in the distribution of regions enriched for RNA Pol II binding in regions showing copy number gain (*P*=0.52) or CNLOH (*P*=0.11) as compared to copy neutral genomic regions. Similarly, we did not observe differences in the distribution of regions enriched for H3K27me3 binding in regions showing gain (*P*=0.61) or CNLOH (*P*=0.89) as compared to copy neutral genomic regions, suggesting that the observed differences in epigenetic marks in the two cell lines are not due to a difference in copy number alterations.

### Overexpression of ALDH1A3 in pancreatic cancer cell lines and tumors

**Figure 3:**
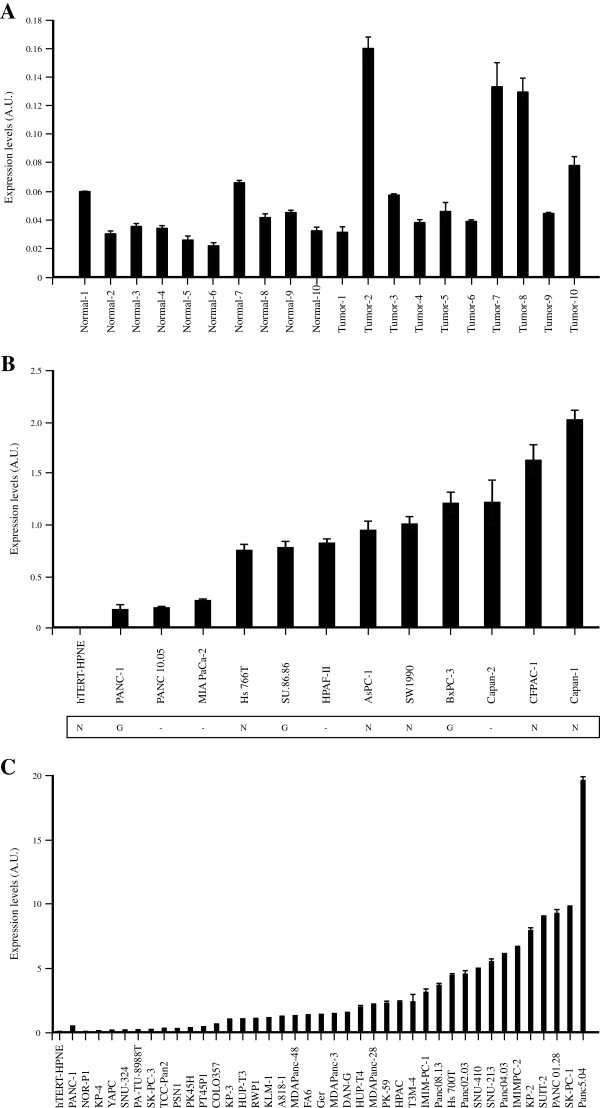
**Expression of the *ALDH1A3 *gene in pancreatic cell lines and tissue samples.** Expression levels for *ALDH1A3* were determined by RT-qPCR in **(A)** normal derived pancreatic tissue samples (N-1 though N-10) and tumor derived pancreatic tissue samples (T1 through T10), as well as **(B)** in pancreatic normal (n=1) and tumor (n=12) derived cell lines. Expression levels were normalized to the geometric mean of *B2M* and *PPIA* and are shown in arbitrary units (A.U.). Chromosomal gain of the *ALDH1A3* gene region is indicated below panel **(B)** as G: copy number gain, N: no gain. Copy number alterations were not assessed for cell lines marked with “–“. **(C)***ALDH1A3* expression levels were assessed in additional pancreatic cancer cell lines (n=39) by RT-qPCR.

We used the transcriptome and epigenome data described above to select genes for assessment of expression differences in independent sets of normal and tumor derived pancreatic tissue samples and cell lines. We based our selection of genes on the strong differences in RNA Pol II and H3K27me3 binding sites in the two cell lines by applying the following criteria: 1.) located within 5 kb of a region enriched ≥ 3 fold for RNA Pol II binding in the PANC-1 cells, 2.) located within 5 kb of a region enriched ≥ 3 fold for H3K27me3 binding in the hTERT-HPNE cells, and 3.) showed ≥ 3 fold increased expression in the PANC-1 as compared to hTERT-HPNE cells. This resulted in a total of 60 genes (labeled in bold in Additional file 4: Table S2). We then selected 4 genes from this list based on varying levels of expression differences and location in genomic regions that either showed copy-number gain (*ALDH1A3*, *SEMA4B*) or did not (*ITGBL1*, *NFE2L3*) in PANC-1 cells. We first assessed gene expression by RT-qPCR in 10 normal and 10 tumor derived pancreatic tissue samples as well as in 11 additional pancreatic cancer cell lines. By selecting differentially expressed genes that were also enriched in positive marks and depleted in negative marks we confirmed differential expression of one of the four genes, *ALDH1A3,* in this set of tumor and normal derived pancreatic tissues and cell lines (Figure 3, panels A and B). This gene was expressed on average 1.93 fold higher in the tumor derived samples as compared to the normal derived pancreatic tissue samples (*P*_*Mann–Whitney U test*_=0.034), and 3 of the 10 tumor samples had over 3 fold higher expression than the average of the normal samples (range 3.32-4.10). The *ALDH1A3* gene was expressed on average 1,308 fold higher in the tumor cell lines as compared to the normal derived hTERT-HPNE cells, and 23.5 fold higher in the tumor cell lines as compared to the average of the normal derived tissue samples. The difference between *ALDH1A3* expression levels in the tumor derived cell lines as compared to the tissue samples may be due to the higher degree of cellular heterogeneity in the latter set. Interestingly, by analyzing copy number alterations, we noted that its expression is increased in cell lines showing copy number gain of the genomic region it resides in (PANC-1, SU.86.86 and BxPC-3) as well as in cell lines without gain (Hs 766T, AsPC-1, Capan-1, SW 1990 and CFPAC-1) as shown in Figure 3B. We confirmed differential expression for the three other genes in the hTERT-HPNE and PANC-1 cell lines by RT-qPCR, but did not observe significantly differential expression in the larger set of cell lines and tissue samples. A second validation in an independent set of 39 additional pancreatic cancer cell lines (Additional file 3: Table S6) showed that *ALDH1A3* was expressed on average 1,630 fold higher in the tumor cell lines as compared to hTERT-HPNE cells, and 78.5 fold higher in the tumor cell lines as compared to the average of the normal derived tissue samples (Figure 3C). Increased RNA Pol II binding was seen in the promoter region of *ALDH1A3* (12.39 fold), and decreased H3K27me3 binding (0.23 fold) in a region overlapping and surrounding exons 7 and 8, in the PANC-1 as compared to the hTERT-HPNE cells (Table 2B). In addition, binding of the negative mark H3K27me3 featured prominently over the first half of the gene in hTERT-HPNE cells (Additional file 10: Figure S4).

## Discussion

Generating global datasets of transcribed sequences and epigenetic marks can form a foundation for genomic and functional investigations of the molecular mechanisms underlying specific cancers. Such datasets have been generated for many tissue types as part of the ENCODE project [30]. To begin to establish genomic datasets for the pancreas we analyzed expressed sequences and signatures of functional elements in normal and pancreatic cancer derived cell lines by massively parallel sequencing. We used a diploid cell line derived from normal pancreatic ducts and immortalized with the *TERT* gene, hTERT-HPNE, and a commonly used hypertriploid pancreatic ductal carcinoma derived cell line, PANC-1, for our studies. In this process, we identified differentially expressed genes that define pathways and cellular processes important for cancer, such as growth factor and receptor activity, signal transduction, focal adhesion, ECM-receptor interaction and cell differentiation. Enrichment of genes in these pathways reiterates the increased growth potential and diminished adhesive properties associated with the development and progression of pancreatic cancer.

Many of the differentially expressed genes encode ligands, receptors and signaling molecules in the wingless and sonic hedgehog signaling pathways, implicated in embryonic development and particular cancers, including that of the pancreas [31-33]. Genes important for focal adhesion and extracellular matrix receptor interaction were also prominent among differentially expressed genes featuring numerous integrin, collagen and laminin subtypes possibly indicating an underlying potential for altered proliferation and interaction with the microenvironment of the tumor derived cell line in an *in vivo* setting. MicroRNA expression has not been assessed before in pancreatic cancer by next generation sequencing methods. Our results in the two cell lines agree with previously published data on the upregulation of *MIR93*, *MIR95*, *MIR135B, MIR181C, MIR181D*, *MIR182, MIR183, MIR190*, *MIR196B* and *MIR203*, and downregulation of *MIR20A* and *MIR29C* in pancreatic intraepithelial neoplasms (PanIns) or pancreatic adenocarcinomas (PDACs) as compared to normal pancreatic tissue [34-36]. In agreement, we noted substantially higher (from 6 to over 200 fold) expression of the former group of genes and lower (3–18 fold) expression of the latter group in our dataset. *MIR135B, MIR182* and *MIR183* are overexpressed in a wide range of other cancer types such as bladder, colon, prostate cancer and glioma [37-41]. In addition, we noted differential expression of miRNA genes not previously reported to be deregulated in cancer. Some of the largest differences were observed for *MIR767* and *MIR1269,* both absent in the hTERT-HPNE cells but highly expressed in the cancer derived PANC-1 cells. The function of *MIR767* and *MIR1269* is largely unknown although the latter may be important during differentiation of human embryonic stem cells [42]. Our results confirm previous findings suggesting that altered miRNA expression contributes to cancerous growth in the pancreas and provide new candidate miRNA genes that require validation in larger sample sets of normal and cancerous pancreatic tissues.

Epigenomic assessment of histone modification patterns revealed an enrichment of mono- and tri-methylated H3K4 in promoters that was highest in actively transcribed genes and gradually lost with lower expression. The transcriptional start site of actively transcribed genes was occupied by RNA polymerase, surrounded by a layer of nucleosomes with tri-methylation at H3K4 and an outer core of nucleosomes with mono-methylation at H3K4. Trimethylation at H3K27 showed an inverse correlation with gene expression. This pattern of modification marks is in accordance with the activating and silencing effects associated with these histone modifications in other cell types [43,44]. Notably, an overall depletion of H3K27me3 marks and enrichment of RNA Pol II sites was seen in the cancer derived cell line, as compared to the normal derived cell line, indicating a possible mechanism of general alleviation of negative epigenetic regulation in pancreatic cancer. By focusing on this apparent difference between epigenetic marks in the two cell lines when selecting genes for further examination, we confirmed higher levels of *ALDH1A3* gene expression in an independent set of tumor derived pancreatic tissues and cell lines in comparison to normal derived pancreatic samples. Of note is that of the genes selected for further assessment, all four showed very similar results in the initial set of cell lines using RT-qPCR, whereas only one showed a similar expression difference in the additional sample sets analyzed. This is probably due to the small initial sample set. Validation in a second larger set of pancreatic cancer cell lines confirmed higher expression levels of *ALDH1A3* as compared to the normal derived cell line and normal derived tissue samples. A limitation of our study is the small number of samples, especially for normal derived pancreatic cell lines and for normal and tumor derived tissue samples.

A general function for members of the aldehyde dehydrogenase (ALDH) superfamily is to serve as detoxification enzymes by converting aldehydes to carboxyl acids (for review see [45]). The different ALDH subfamilies and their members have additional catalytic functions; the ALDH1A family is responsible for the conversion of retinaldehyde to retinoic acid (RA) and its three members (ALDH1A1 through ALD1A3) are thus important regulators of RA signaling. Increased aldehyde dehydrogenase activity is commonly seen in many cancer types where it appears to specifically mark cancer stem cells [46,47]. These cells tend to be highly tumorigenic and invasive, and are often resistant to conventional therapy [48]. The presence of aldehyde dehydrogenase positive cells in tumor from patients with pancreatic cancer has been associated with decreased survival, and ALDH1A1 expression (ALDH1A3 was not tested) correlated with invasion of pancreatic cancer cell lines [49]. Of the 19 ALDH isoforms, ALDH1A1 has most often been linked to the increased ALDH activity of cancer stem cells [50]. However, ALDH1A3 expression has been shown to correlate better with aldehyde activity in breast cancer stem cells than ALDH1A1 expression, and correlate with tumor grade, stage and metastasis [51]. Although we confirmed increased mRNA levels of *ALDH1A3* in an independent set of pancreatic tissue samples and two series of cell lines, our finding should be validated in additional sample sets to thoroughly investigate the relationship between ADH1A3, cancer stem cells and pancreatic cancer.

## Conclusion

The high resolution and read depth of next generation sequencing has allowed us to accurately quantify gene expression and locate epigenetic marks, thus enabling a detailed analysis of the pancreatic cancer genome. Although limited by a small set of samples, this study has thoroughly catalogued the transcriptome and epigenome of two pancreatic cell lines, confirmed previous genes and pathways deregulated in pancreatic cancer and generated a series of hypotheses worth following-up in future studies to investigate key events in pancreatic carcinogenesis.

## Competing interest

The authors report no financial interests or potential conflicts of interests.

## Authors’ contributions

JJ, HPa, JH and LA conceived the study; JJ and HPa performed the analysis of all datasets; WX, HPa, XL and JP performed alignment of next generation sequencing datasets; IC and HPf performed cell culture, RNA and DNA isolation; JJ performed RT-qPCR experiments; WZ and ZW performed CNV analysis; HPf and UR provided cell lines, DNA and RNA; GMP provided tissue samples; JJ, HPa, JH, HPf, SST, GMP and LA wrote the manuscript. All authors read and approved the final manuscript.

## Pre-publication history

The pre-publication history for this paper can be accessed here:

http://www.biomedcentral.com/1755-8794/6/33/prepub

## Supplementary Material

Additional file 1: Table S1 Number of reads generated by mRNA-seq (A), miRNA-Seq (B), and ChIP-seq (C) experiments and their alignment characteristics. The number of peaks from each ChIP-seq experiment is shown in (C). Click here for file

Additional file 2: Figure S1 RNA-seq analysis pipeline for read alignment and digital gene expression quantification. Click here for file

Additional file 3: Table S6 Additional cell lines used for *ALDH1A3* expression assessment by RT-qPCR. Names of pancreatic cancer cell lines, references and additional information on how they were obtained. Click here for file

Additional file 4: Table S2 Differentially expressed mRNA genes in tumor and normal derived pancreatic cell lines. mRNA genes with a 3 fold or greater expression difference (n=1,983) are listed, as are expression values in RPKM and FDR values. Genes that are expressed ≥3 fold higher in the PANC-1 cells, located within 5 kb of regions enriched ≥3 fold in RNA Pol II binding in the PANC-1 cells, and enriched ≥3 fold in H3K27me3 binding in the hTERT-HPNE cells are marked in bold text. Click here for file

Additional file 5: Table S3 Differentially expressed miRNA genes in tumor and normal derived pancreatic cell lines. miRNA genes with a 3 fold or greater expression difference (n=163) are listed, as are expression values in RPKM and FDR values. Click here for file

Additional file 6: Table S4 GO analysis for enrichment of biological processes (GO BP) for mRNA and miRNA genes differentially expressed in tumor and normal derived pancreatic cell lines. Statistical significance is shown by *P* values and FDR values. Click here for file

Additional file 7: Table S5 GO analysis for enrichment of molecular function (GO MF) for mRNA and miRNA genes differentially expressed in tumor and normal derived pancreatic cell lines. Statistical significance is shown by *P* values and FDR values. Click here for file

Additional file 8: Figure S2 Distribution of histone modification peaks and RNA Pol II binding sites according to locations within promoters, regions downstream of genes, 5′ and 3′ untranslated regions (UTR), coding exons, introns and distal intergenic regions of the genome for the hTERT-HPNE (A) and PANC-1 (B) cells by analysis with the CEAS software. Click here for file

Additional file 9: Figure S3 Distribution of histone modification marks and RNA Polymerase II binding sites according to gene expression levels for RefSeq genes in the hTERT-HPNE cell line. Genes were divided into four categories according to gene expression values (RPKM) from high (A), medium (B), low (C) to very low (D). Density traces for histone modification sequence tags were then graphed according to the start of transcription (TSS) for each of the four groups. Density traces are labeled in green for H3K4me1, red for H3K4me3, blue for H3K27me3 and purple for Pol II. Click here for file

Additional file 10: Figure S4 Distribution of sequence reads for RNA Pol II and H3K27me3 in the *ALDH1A3* gene in PANC-1 and hTERT-HPNE cells. Note increased RNA Pol II binding in the promoter of *ALDH1A3* in PANC-1 cells, and increased H3K27me3 binding in first half of the gene in the hTERT-HPNE cells. Click here for file

## References

[B1] Jemal A Siegel R Xu J Ward E Cancer statistics, 2010 CA Cancer J Clin 2010 60 5 277 300 10.3322/caac.20073 20610543

[B2] Ferlay J Bray F Pisani P Parkin DM GLOBOCAN 2002: cancer incidence, mortality and prevalence worldwide 2004 Lyon: IARC Press

[B3] Scarlett CJ Salisbury EL Biankin AV Kench J Precursor lesions in pancreatic cancer: morphological and molecular pathology Pathology 2011 43 3 183 200 10.1097/PAT.0b013e3283445e3a 21436628

[B4] Jones S Zhang X Parsons DW Lin JC Leary RJ Angenendt P Mankoo P Carter H Kamiyama H Jimeno A Core signaling pathways in human pancreatic cancers revealed by global genomic analyses Science 2008 321 5897 1801 1806 10.1126/science.1164368 18772397 PMC2848990

[B5] Campbell PJ Yachida S Mudie LJ Stephens PJ Pleasance ED Stebbings LA Morsberger LA Latimer C McLaren S Lin ML The patterns and dynamics of genomic instability in metastatic pancreatic cancer Nature 2010 467 7319 1109 1113 10.1038/nature09460 20981101 PMC3137369

[B6] Gronbaek K Hother C Jones PA Epigenetic changes in cancer APMIS 2007 115 10 1039 1059 10.1111/j.1600-0463.2007.apm_636.xml.x 18042143

[B7] Herman JG Baylin SB Gene silencing in cancer in association with promoter hypermethylation N Engl J Med 2003 349 21 2042 2054 10.1056/NEJMra023075 14627790

[B8] Jenuwein T Allis CD Translating the histone code Science 2001 293 5532 1074 1080 10.1126/science.1063127 11498575

[B9] Omura N Li CP Li A Hong SM Walter K Jimeno A Hidalgo M Goggins M Genome-wide profiling of methylated promoters in pancreatic adenocarcinoma Cancer Biol Ther 2008 7 7 1146 1156 10.4161/cbt.7.7.6208 18535405 PMC2763640

[B10] Sato N Goggins M The role of epigenetic alterations in pancreatic cancer J Hepatobiliary Pancreat Surg 2006 13 4 286 295 10.1007/s00534-005-1057-1 16858539

[B11] Vincent A Omura N Hong SM Jaffe A Eshleman J Goggins M Genome-wide analysis of promoter methylation associated with gene expression profile in pancreatic adenocarcinoma Clinical cancer research: an official journal of the American Association for Cancer Research 2011 17 13 4341 4354 10.1158/1078-0432.CCR-10-3431 21610144 PMC3131423

[B12] Lee KM Yasuda H Hollingsworth MA Ouellette MM Notch 2-positive progenitors with the intrinsic ability to give rise to pancreatic ductal cells Laboratory investigation; a journal of technical methods and pathology 2005 85 8 1003 1012 10.1038/labinvest.3700298 15924149

[B13] Lee KM Nguyen C Ulrich AB Pour PM Ouellette MM Immortalization with telomerase of the Nestin-positive cells of the human pancreas Biochem Biophys Res Commun 2003 301 4 1038 1044 10.1016/S0006-291X(03)00086-X 12589817

[B14] Lieber M Mazzetta J Nelson-Rees W Kaplan M Todaro G Establishment of a continuous tumor-cell line (panc-1) from a human carcinoma of the exocrine pancreas Int J Cancer 1975 15 5 741 747 10.1002/ijc.2910150505 1140870

[B15] Li H Durbin R Fast and accurate short read alignment with Burrows-Wheeler transform Bioinformatics 2009 25 14 1754 1760 10.1093/bioinformatics/btp324 19451168 PMC2705234

[B16] Larsson TP Murray CG Hill T Fredriksson R Schioth HB Comparison of the current RefSeq, Ensembl and EST databases for counting genes and gene discovery FEBS Lett 2005 579 3 690 698 10.1016/j.febslet.2004.12.046 15670830

[B17] Mortazavi A Williams BA McCue K Schaeffer L Wold B Mapping and quantifying mammalian transcriptomes by RNA-Seq Nat Methods 2008 5 7 621 628 10.1038/nmeth.1226 18516045 PMC13303166

[B18] Robinson MD McCarthy DJ Smyth GK edgeR: a bioconductor package for differential expression analysis of digital gene expression data Bioinformatics 2010 26 1 139 140 10.1093/bioinformatics/btp616 19910308 PMC2796818

[B19] Ronen R Gan I Modai S Sukacheov A Dror G Halperin E Shomron N miRNAkey: a software for microRNA deep sequencing analysis Bioinformatics 2010 26 20 2615 2616 10.1093/bioinformatics/btq493 20801911

[B20] Kozomara A Griffiths-Jones S miRBase: integrating microRNA annotation and deep-sequencing data Nucleic Acids Res 2011 39 D152 157 10.1093/nar/gkq1027 21037258 PMC3013655

[B21] Kanehisa M Goto S KEGG: kyoto encyclopedia of genes and genomes Nucleic Acids Res 2000 28 1 27 30 10.1093/nar/28.1.27 10592173 PMC102409

[B22] Ashburner M Ball CA Blake JA Botstein D Butler H Cherry JM Davis AP Dolinski K Dwight SS Eppig JT Gene ontology: tool for the unification of biology: the gene ontology consortium Nat Genet 2000 25 1 25 29 10.1038/75556 10802651 PMC3037419

[B23] Young MD Wakefield MJ Smyth GK Oshlack A Gene ontology analysis for RNA-seq: accounting for selection bias Genome biology 2010 11 2 R14 10.1186/gb-2010-11-2-r14 20132535 PMC2872874

[B24] Langmead B Trapnell C Pop M Salzberg SL Ultrafast and memory-efficient alignment of short DNA sequences to the human genome Genome biology 2009 10 3 R25 10.1186/gb-2009-10-3-r25 19261174 PMC2690996

[B25] Zang C Schones DE Zeng C Cui K Zhao K Peng W A clustering approach for identification of enriched domains from histone modification ChIP-Seq data Bioinformatics 2009 25 15 1952 1958 10.1093/bioinformatics/btp340 19505939 PMC2732366

[B26] Shin H Liu T Manrai AK Liu XS CEAS: cis-regulatory element annotation system Bioinformatics 2009 25 19 2605 2606 10.1093/bioinformatics/btp479 19689956

[B27] Pique-Regi R Caceres A Gonzalez JR R-Gada: a fast and flexible pipeline for copy number analysis in association studies BMC bioinformatics 2010 11 380 10.1186/1471-2105-11-380 20637081 PMC2915992

[B28] Jacobs KB Yeager M Zhou W Wacholder S Wang Z Rodriguez-Santiago B Hutchinson A Deng X Liu C Horner MJ Detectable clonal mosaicism and its relationship to aging and cancer Nat Genet 2012 44 6 651 658 10.1038/ng.2270 22561519 PMC3372921

[B29] Guescini M Sisti D Rocchi MB Stocchi L Stocchi V A new real-time PCR method to overcome significant quantitative inaccuracy due to slight amplification inhibition BMC bioinformatics 2008 9 326 10.1186/1471-2105-9-326 18667053 PMC2533027

[B30] Raney BJ Cline MS Rosenbloom KR Dreszer TR Learned K Barber GP Meyer LR Sloan CA Malladi VS Roskin KM ENCODE whole-genome data in the UCSC genome browser (2011 update) Nucleic Acids Res 2011 39 D871 875 10.1093/nar/gkq1017 21037257 PMC3013645

[B31] Humke EW Dorn KV Milenkovic L Scott MP Rohatgi R The output of hedgehog signaling is controlled by the dynamic association between suppressor of fused and the Gli proteins Genes Dev 2010 24 7 670 682 10.1101/gad.1902910 20360384 PMC2849124

[B32] Lee DY Deng Z Wang CH Yang BB MicroRNA-378 promotes cell survival, tumor growth, and angiogenesis by targeting SuFu and Fus-1 expression Proc Natl Acad Sci USA 2007 104 51 20350 20355 10.1073/pnas.0706901104 18077375 PMC2154434

[B33] Morris JP Wang SC Hebrok M KRAS, hedgehog, Wnt and the twisted developmental biology of pancreatic ductal adenocarcinoma Nat Rev Cancer 2010 10 10 683 695 20814421 10.1038/nrc2899 PMC4085546

[B34] Park JY Helm J Coppola D Kim D Malafa M Kim SJ MicroRNAs in pancreatic ductal adenocarcinoma World J Gastroenterol 2011 17 7 817 827 10.3748/wjg.v17.i7.817 21412491 PMC3051132

[B35] Yu J Li A Hong SM Hruban RH Goggins M MicroRNA alterations of pancreatic intraepithelial neoplasms (PanINs) Clinical cancer research 2012 18 4 981 992 10.1158/1078-0432.CCR-11-2347 22114139 PMC3288338

[B36] Munding JB Adai AT Maghnouj A Urbanik A Zollner H Liffers ST Chromik AM Uhl W Szafranska-Schwarzbach AE Tannapfel A Global microRNA expression profiling of microdissected tissues identifies miR-135b as a novel biomarker for pancreatic ductal adenocarcinoma International journal of cancer 2011 131 2 E86 E95 10.1002/ijc.26466 21953293

[B37] Han Y Chen J Zhao X Liang C Wang Y Sun L Jiang Z Zhang Z Yang R Li Z MicroRNA expression signatures of bladder cancer revealed by deep sequencing PloS one 2011 6 3 e18286 10.1371/journal.pone.0018286 21464941 PMC3065473

[B38] Bandres E Cubedo E Agirre X Malumbres R Zarate R Ramirez N Abajo A Navarro A Moreno I Monzo M Identification by real-time PCR of 13 mature microRNAs differentially expressed in colorectal cancer and non-tumoral tissues Mol Cancer 2006 5 29 16854228 10.1186/1476-4598-5-29 PMC1550420

[B39] Jiang L Mao P Song L Wu J Huang J Lin C Yuan J Qu L Cheng SY Li J miR-182 as a prognostic marker for glioma progression and patient survival Am J Pathol 2010 177 1 29 38 10.2353/ajpath.2010.090812 20472885 PMC2893648

[B40] Schaefer A Jung M Mollenkopf HJ Wagner I Stephan C Jentzmik F Miller K Lein M Kristiansen G Jung K Diagnostic and prognostic implications of microRNA profiling in prostate carcinoma International journal of cancer Journal international du cancer 2010 126 5 1166 1176 19676045 10.1002/ijc.24827

[B41] Necela BM Carr JM Asmann YW Thompson EA Differential expression of microRNAs in tumors from chronically inflamed or genetic (APC(Min/+)) models of colon cancer PloS one 2011 6 4 e18501 10.1371/journal.pone.0018501 21532750 PMC3075242

[B42] Morin RD O’Connor MD Griffith M Kuchenbauer F Delaney A Prabhu AL Zhao Y McDonald H Zeng T Hirst M Application of massively parallel sequencing to microRNA profiling and discovery in human embryonic stem cells Genome Res 2008 18 4 610 621 10.1101/gr.7179508 18285502 PMC2279248

[B43] Heintzman ND Stuart RK Hon G Fu Y Ching CW Hawkins RD Barrera LO Van Calcar S Qu C Ching KA Distinct and predictive chromatin signatures of transcriptional promoters and enhancers in the human genome Nat Genet 2007 39 3 311 318 10.1038/ng1966 17277777

[B44] Barski A Cuddapah S Cui K Roh TY Schones DE Wang Z Wei G Chepelev I Zhao K High-resolution profiling of histone methylations in the human genome Cell 2007 129 4 823 837 10.1016/j.cell.2007.05.009 17512414

[B45] Marchitti SA Brocker C Stagos D Vasiliou V Non-P450 aldehyde oxidizing enzymes: the aldehyde dehydrogenase superfamily Expert opinion on drug metabolism & toxicology 2008 4 6 697 720 10.1517/17425255.4.6.697 18611112 PMC2658643

[B46] Deng S Yang X Lassus H Liang S Kaur S Ye Q Li C Wang LP Roby KF Orsulic S Distinct expression levels and patterns of stem cell marker, aldehyde dehydrogenase isoform 1 (ALDH1), in human epithelial cancers PloS one 2010 5 4 e10277 10.1371/journal.pone.0010277 20422001 PMC2858084

[B47] Ginestier C Hur MH Charafe-Jauffret E Monville F Dutcher J Brown M Jacquemier J Viens P Kleer CG Liu S ALDH1 is a marker of normal and malignant human mammary stem cells and a predictor of poor clinical outcome Cell stem cell 2007 1 5 555 567 10.1016/j.stem.2007.08.014 18371393 PMC2423808

[B48] Li Y Kong D Ahmad A Bao B Sarkar FH Pancreatic cancer stem cells: emerging target for designing novel therapy Cancer letters 2013 338 1 94 100 10.1016/j.canlet.2012.03.018 22445908 PMC3399061

[B49] Rasheed ZA Yang J Wang Q Kowalski J Freed I Murter C Hong SM Koorstra JB Rajeshkumar NV He X Prognostic significance of tumorigenic cells with mesenchymal features in pancreatic adenocarcinoma J Natl Cancer Inst 2010 102 5 340 351 10.1093/jnci/djp535 20164446 PMC2831049

[B50] Marcato P Dean CA Giacomantonio CA Lee PW Aldehyde dehydrogenase: its role as a cancer stem cell marker comes down to the specific isoform Cell Cycle 2011 10 9 1378 1384 10.4161/cc.10.9.15486 21552008

[B51] Marcato P Dean CA Pan D Araslanova R Gillis M Joshi M Helyer L Pan L Leidal A Gujar S Aldehyde dehydrogenase activity of breast cancer stem cells is primarily due to isoform ALDH1A3 and its expression is predictive of metastasis Stem Cells 2011 29 1 32 45 10.1002/stem.563 21280157

